# A tricarboxylic acid cycle-based machine learning model to select effective drug targets for the treatment of esophageal squamous cell carcinoma

**DOI:** 10.3389/fphar.2023.1195195

**Published:** 2023-06-13

**Authors:** Yicheng Liang, Binghua Tan, Minjun Du, Bing Wang, Yushun Gao, Minghui Wang

**Affiliations:** ^1^ Department of Thoracic Surgery, Sun Yat-sen Memorial Hospital, Sun Yat-sen University, Guangzhou, China; ^2^ Guangdong Provincial Key Laboratory of Malignant Tumor Epigenetics and Gene Regulation, Sun Yat-sen Memorial Hospital, Sun Yat-sen University, Guangzhou, China; ^3^ Department of Thoracic Surgery, National Cancer Center/National Clinical Research Center for Cancer/Cancer Hospital, Chinese Academy of Medical Sciences and Peking Union Medical College, Beijing, China

**Keywords:** esophageal squamous carcinoma, tricarboxylic acid cycle, imune infiltration, tumour microenvironment, prognostic markers, selection of drugs

## Abstract

**Background:** The tricarboxylic acid cycle (TCA cycle) is an important metabolic pathway and closely related to tumor development. However, its role in the development of esophageal squamous cell carcinoma (ESCC) has not been fully investigated.

**Methods:** The RNA expression profiles of ESCC samples were retrieved from the TCGA database, and the GSE53624 dataset was additionally downloaded from the GEO database as the validation cohort. Furthermore, the single cell sequencing dataset GSE160269 was downloaded. TCA cycle-related genes were obtained from the MSigDB database. A risk score model for ESCC based on the key genes of the TCA cycle was built, and its predictive performance was evaluated. The association of the model with immune infiltration and chemoresistance were analyzed using the TIMER database, the R package “oncoPredict” score, TIDE score and so on. Finally, the role of the key gene CTTN was validated through gene knockdown and functional assays.

**Results:** A total of 38 clusters of 8 cell types were identified using the single-cell sequencing data. The cells were divided into two groups according to the TCA cycle score, and 617 genes were identified that were most likely to influence the TCA cycle. By intersecting 976 key genes of the TCA cycle with the results of WGCNA, 57 genes significantly associated with the TCA cycle were further identified, of which 8 were screened through Cox regression and Lasso regression to construct the risk score model. The risk score was a good predictor of prognosis across subgroups of age, N, M classification and TNM stage. Furthermore, BI-2536, camptothecin and NU7441 were identified as possible drug candidates in the high-risk group. The high-risk score was associated with decreased immune infiltration in ESCC, and the low-risk group had better immunogenicity. In addition, we also evaluated the relationship between risk scores and immunotherapy response rates. Functional assays showed that CTTN may affect the proliferation and invasion of ESCC cells through the EMT pathway.

**Conclusion:** We constructed a predictive model for ESCC based on TCA cycle-associated genes, which achieved good prognostic stratification. The model are likely associated with the regulation of tumor immunity in ESCC.

## Introduction

Esophageal cancer is one of the leading causes of cancer-related deaths worldwide. According to the Global Cancer Statistics 2020, esophageal cancer ranks 7th and 6th in terms of incidence and mortality (Chen et al., 2016; Arnold et al., 2020; Sung et al., 2021). The 5-year survival rate for patients with resectable esophageal cancer is close to 30%, while unresectable tumors are mainly treated with radiotherapy and chemotherapy, with a median survival of only 17–54 months (Cookson, 2000; Pape et al., 2022). Thus, it is crucial to identify novel therapeutic targets of ESCC in order to improve prognosis.

The tricarboxylic acid cycle (TCA cycle) is a crucial pathway for the metabolism of sugars, lipids, and amino acids (Fernie et al., 2004; Salway, 2018), and its key enzymes are succinate dehydrogenase (SDH), ferredoxin hydratase (FH), and isocitrate dehydrogenase (IDH) (Eniafe and Jiang, 2021). In the last decade, several dominant mutations in genes encoding the mitochondrial and cytoplasmic subtypes of SDH, FH and IDH have been identified that are associated with tumorigenesis (Kaelin and McKnight, 2013; Laurenti and Tennant, 2016). Furthermore, previous study showed that TCA cycle played an important role in promoting cancer metastasis (Cai et al., 2020). Therefore, it is worth investigating the role of the TCA cycle in tumorigenesis. However, any specific association between TCA cycle-related genes and ESCC development has not been demonstrated.

Given the significance of the TCA cycle and the metabolic dysfunction that occurs during tumor development, we used an innovative bioinformatics approach to screen for the key TCA-related genes involved in ESCC in order to identify potential therapeutic targets. In addition, we established a risk score model based on the prognostically significant TCA cycle-related genes to identify patients with poor prognosis and monitor therapeutic response.

## Methods

### Data acquisition

The RNA expression profiles of ESCC were obtained from The Cancer Genome Atlas (TCGA) database. The samples were subsequently split into a training and validation cohort at 5:5 ratio. The GEO dataset (GSE53624) was downloaded as an independent validation cohort. All data were in FPKM format and converted to log2 scale. The R package “limma” was used to adjust the batch effects between TCGA and GSE53624. In addition to DNA expression, DNA methylation, IncRNA and mRNA expression data, clinical information (pathological type, tumor location, tumor stage, age, ethnicity, survival status, etc.) was also extracted from the datasets. The single-cell sequencing dataset GSE160269 that includes 60 samples was obtained from the GEO database. All datasets were required to meet the following analysis criteria: 1) availability of complete information, 2) sample size >50, and 3) all cases underwent surgery without prior radiotherapy or other treatment.

### Annotation of major cell types in ESCC

The quality control of the scRNA-seq data was performed using the “seurat” R package. The TCA cycle-related genes were extracted from the MSigDB database. To assign a TCA cycle activity score to each cell type, the “AUCell R” package was used to determine the status of gene set activity. The TCA cycle score for each patient in TCGA cohort was determined by Single sample gene set enrichment analysis (ssGSEA). Finally, weighted correlation network analysis (WGCNA) was performed to derive gene clusters that were correlated with TCA cycle scores using transcriptomic data of ESCC.

### Construction and evaluation of TCA cycle gene-based risk model

The TCA cycle genes significantly associated with survival in ESCC were first identified through univariate Cox analysis, with *p* < 0.05 as the threshold. Lasso regression was then performed on the survival-associated genes to eliminate covariates, and the best gene combination was determined to construct a risk score prediction model. The risk scores for all patients in the training cohort were calculated, and the patients were stratified into the high-and low-risk groups using the median risk score as the threshold value. The survival curves of two groups were plotted using the Kaplan-Meier method, and compared with the log-rank test to assess the predictive effect of the model. The prognostic model was similarly tested on multiple validation cohorts. The predictive performance of the prognostic model was further assessed by plotting ROC curves at different endpoints and calculating the area under the curve (AUC). A nomogram was then constructed by combining the TCA risk score and other clinical parameters, and its predictive performance was evaluated by the Kaplan-Meier method and log-rank test as described. The clinical practicability of the nomogram was assessed by decision curve analysis (DCA). Stratified analyses were also performed for subgroups based on different clinical features.

### Immune infiltration analysis and drug-sensitivity analysis

The level of immune infiltration in ESCC patients was determined by seven algorisms, including CIBERSORT, CIBERSORT_ABS, EPIC, MCPcounter, quanTIseq, TIMER, xCell. In addition, ssGSEA was performed on genes in the TCA-related model. The “estimate” R package was used to determine the relative abundance of stromal cells, immune cells, and tumor cells, and compare these values across risk categories. The genetic mutations in the high-risk and low-risk patients were also compared by analyzing the mutation profiles of ESCC patients from TCGA using the “maftools” software. The half-maximal inhibitory concentration (IC50) of common chemotherapeutic agents in the two risk groups using the R package “oncoPredict”.

### Cell culture and siRNA transfection

Two esophageal cancer cell lines, KYSE150 and TE-1, were procured from the American Type Culture Collection (ATCC) repository. All cell lines were cultured in RPMI medium at 37°C in a humidified incubator containing 5% CO_2_. CTTN was knocked down in both cell lines using two specific siRNAs (si-CTTN-1, si-CTTN-2) that were synthesized by GenePharma (Shanghai, China). The cells were transfected with the siRNA constructs using JetPRIME (PolyPlus transfections SA, Illkirch, France) according to the manufacturer’s instructions, and harvested 48 h later for follow-up experiments.

### Quantitative reverse transcription PCR (RT-qPCR)

Total RNA was extracted from the cells using TRIzol reagent (Invitrogen, United States), and reverse transcribed using PrimeScript RT Master Mix (Takara, Japan). RT-PCR was performed using TB Green Premix Ex Taq II (Tli RNaseH Plus) (Takara, Japan) on the ABI 7500 qPCR cycler (Applied Biosystems, United States). The primers were synthesized by Sangon Biotech (Shanghai), and the sequences were as follows: CTTN forward, 5′-GTG​GTT​TTG​GCG​GCA​AGT​ATG-3′; CTTN reverse, 5′-CTC​TCT​GTG​ACT​CGT​GCT​TCT-3′; GAPDH forward, 5′-CGA​GAT​CCC​TCC​AAA​ATC​AA-3′, GAPDH reverse, 5′-TTC​ACA​CCC​ATG​ACG​AAC​AT-3′; E-cadherin forward, 5′-ATT​TTT​CCC​TCG​ACA​CCC​GAT-3′; E-cadherin reverse, 5′-TCC​CAG​GCG​TAG​ACC​AAG​A-3′; Vimentin forward, 5′-AGT​CCA​CTG​AGT​ACC​GGA​GAC-3′; Vimentin reverse, 5′-CAT​TTC​ACG​CAT​CTG​GCG​TTC-3′; N-cadherin forward, 5′-ATT​GGA​CCA​TCA​CTC​GGC​TTA-3′; N-cadherin reverse 5′-CAC​ACT​GGC​AAA​CCT​TCA​CG-3′; Snail forward, 5′-TCG​GAA​GCC​TAA​CTA​CAG​CGA-3′; Snail reverse, 5′-TCG​GAA​GCC​TAA​CTA​CAG​CGA-3′. Each reaction was performed in triplicate.

### EDU assay

The cells transfected with si-NC, si-CTTN-1 or si-CTTN-2 were seeded in a 24-well plate at the density of 6 × 10^4^ cells/well, and labelled using the BeyoClick™ EdU-488 Cell Proliferation Assay Kit after 24 h of culture. The fluorescence intensity of the cells was measured, and the ratio of EdU-488+ cells to DAPI+ cells was calculated to determine the proliferative capacity.

### Clone formation assay

KYSE150 and TE-1 cells were seeded in 6-well plates at the logarithmic growth stage, and the culture medium was replaced every 3 days. Following transfection, the cells were cultured for 12 days. The resulting colonies were fixed with 4% paraformaldehyde, stained with 0.1% crystal violet, and counted.

### Transwell assay

The suitably transfected cells were seeded into the upper chamber of Transwell inserts pre-coated with 1:5 diluted Matrigel in serum-free medium. The lower chambers were filled with complete medium. After culturing for 24 h, the cells remaining on the upper surface were swabbed off, and those that migrated to the lower surface were fixed with 4% paraformaldehyde and stained with 0.1% crystal violet. The number of cells were counted in 5 random fields of view per well to determine migration rate.

### Western blotting

Proteins isolated from the transfected cells were separated by SDS-PAGE and transferred to PVDF membranes. After blocking with 5% skimmed milk powder for 2 h, the membranes were incubated overnight with primary antibodies specific for GAPDH (ab9485, Abcam; dilution: 1:2500), E-cadherin (ab40772, Abcam; dilution: 1:2000), Snail (ab216347, Abcam; dilution: 1:1000), Vimentin (ab92547, Abcam; dilution: 1:2000) and N-cadherin (ab76011, Abcam; dilution: 1:5000) at 4°C. The membranes were washed thrice with TBST for 10 min each time, and then incubated for 1 h with peroxidase-conjugated secondary antibody (ab6721, Abcam; dilution: 1:2000).

## Results

### Single-cell analysis and functional annotation of ESCC samples

**FIGURE 1:**
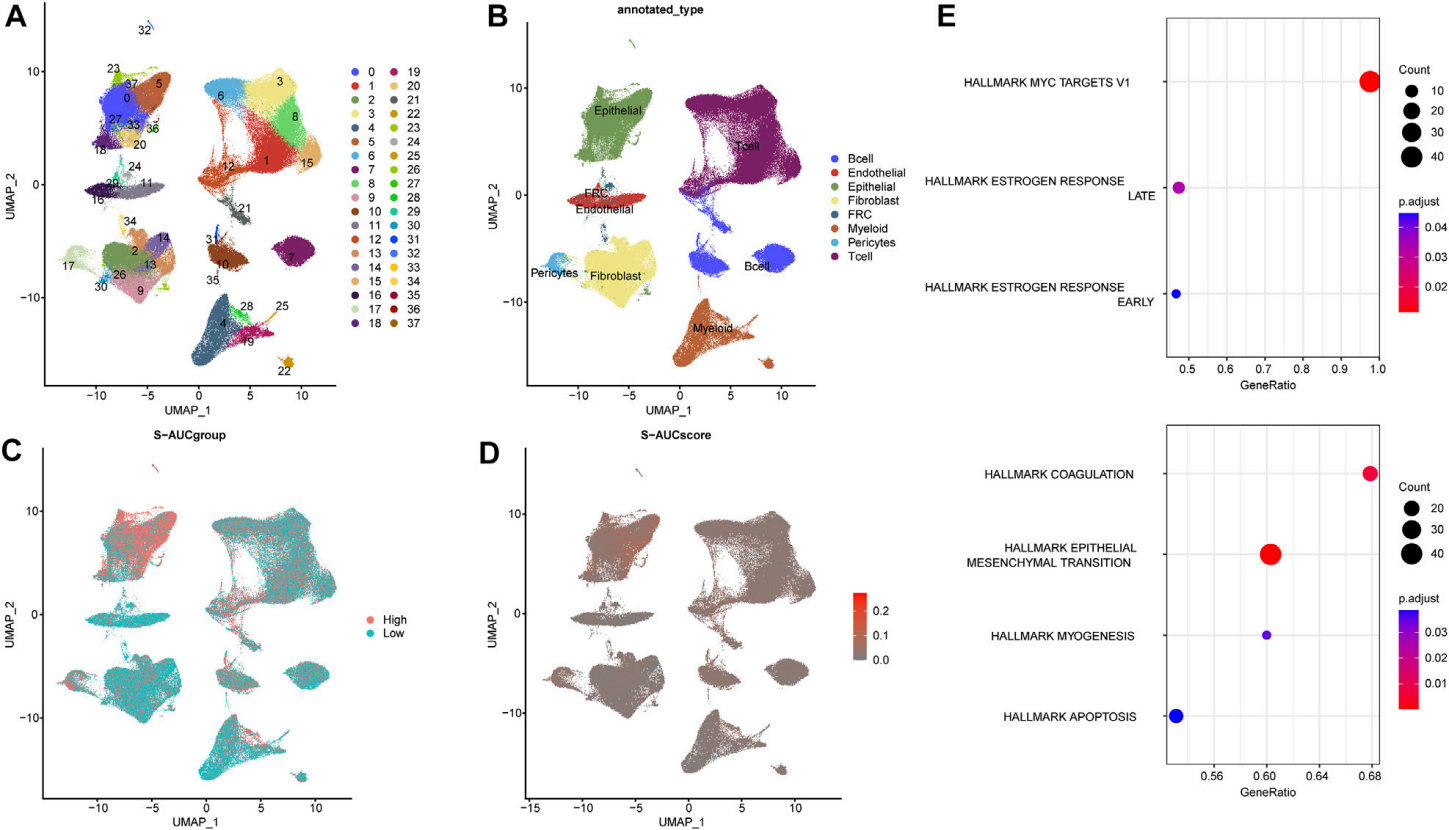
Single-cell sequencing data analysis and annotation. **(A)** UMAP downscaling of ESCC single cell samples into 38 detailed cell clusters. **(B)** Annotation of 8 cell types, including T cells, B cells, endothelial cells, epithelial cells, myeloid cells, fibroblast cells, fibroblastic reticular cells (FRC), and pericytes. **(C)** The high and low subgroups divided by AUC scores of cell populations. **(D)** AUC scores in ESCC annotated with the results of the different cell types. **(E)** Biological function enrichment of TCA cycle-related differential genes.

Using the UMAP method, we annotated the ESCC single cell samples into 38 clusters (Figure 1A) and eight distinct cell types, including T cells, B cells, endothelial cells, epithelial cells, fibroblasts, fibroblastic reticular cells (FRC), myeloid cells and pericytes. The T cells, fibroblasts and epithelial cells were the predominant cell types, whereas pericytes and FRCs were relatively less abundant (Figure 1B). The AUCell R package was used to determine the TCA cycling activity in the individual cells, which were then stratified into the high and low TCA cycle groups based on the AUC scores. The epithelial and myeloid cells were the predominant cell types with high expression of TCA-related genes (Figures 1C, D). To elucidate the potential biological mechanisms, we screened for the differentially expressed genes (DEGs) and pathways associated with the TCA cycle between the high and low TCA-AUC subgroups. A total of 617 genes were identified that are most likely to influence the TCA cycle, and showed significant enrichment of tumor-associated pathways such as Myc targets v1, coagulation, estrogen response, EMT, and myogenesis (Figure 1E).

**FIGURE 2:**
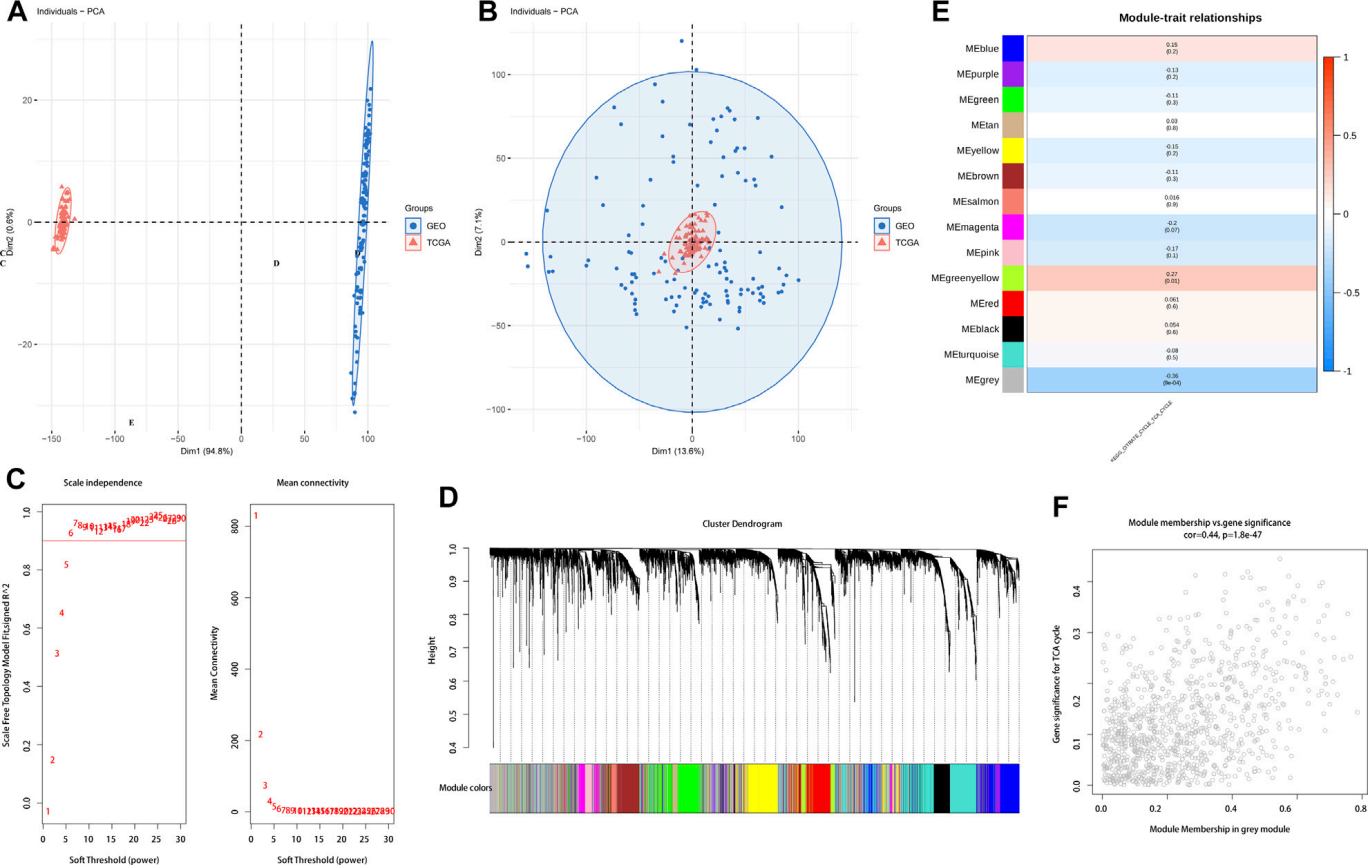
Distribution of TCA cycle-related genes. **(A)**. PCA of the TCGA and GEO datasets before batch processing. **(B)** PCA of the TCGA and GEO datasets after batch processing. **(C)** Network topology analysis of different soft threshold powers. **(D)** Hierarchical clustering dendrogram of co-expressed genes identified by ESCC modules. **(E)** Module feature genes associated with TCA cycle-related module in ESCC. **(F)** Enrichment of TCA cycle-related DEGs for grey modules.

**FIGURE 3:**
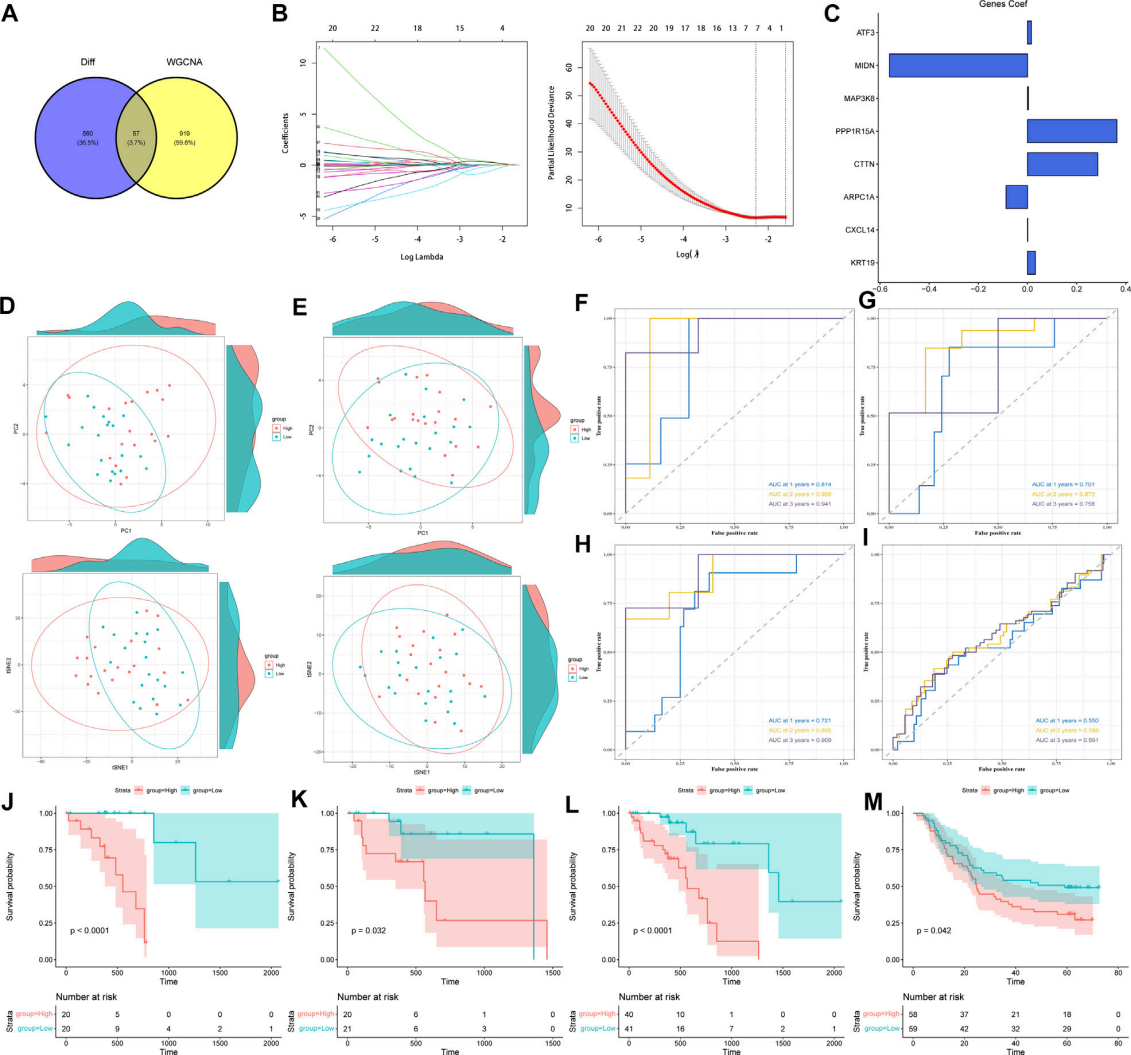
Construction and validation of TCA cycle-related risk score model. **(A)** Intersection of the most relevant genes affecting TCA cycle activity in single cells and the differential genes identified by WGCNA. **(B)** Screening of genes for constructing TCA cycle risk score model by LASSO Cox regression. **(C)** The weights of the eight genes constituting the TCA risk score model. **(D)** PCA and t-SNE analysis in the training cohort. **(E)** PCA and t- SNE analysis in the validation cohort. **(F–H)** ROC curves of the TCA cycle risk score model for predicting 1-year, 2-year, and 3-year survival in the TCGA training cohort **(F)**, validation cohort **(G)**, and complete cohort **(H)**. **(I)** ROC curves of the TCA cycle risk score model for predicting 1-, 2-, and 3-year survival in the GSE53624 dataset. **(J–M)** Survival curves of the high-risk and low-risk groups in the **(J)** TCGA training cohort, **(K)** TCGA validation cohort, **(L)** complete cohort, and **(M)** GSE53624 cohort.

The differential genes obtained from the TCGA cohort were used for WGCNA after logarithmic transformation and removal of genes with missing values. Principal component analysis of the samples with clinical features indicated that the TCGA and GSE53624 cohorts were independent, with significant batch effects (Figure 2A). To further elucidate the correlation between the TCA cycle and ESCC microenvironment, we performed de-batch effect manipulation. As shown in Figure 2B, elimination of batch effects resulted in enhanced accuracy. Furthermore, we constructed a gene co-expression network with optimal soft threshold P (weighing parameter) set to 6, which ensured scale-free distribution (Figure 2C). Modules with similarity less than 12 were merged and the minimum number of modules was set to 30 (Figure 2D). As shown in Figures 2E, F, the grey modules contained 976 genes, and were most closely associated with the TCA cycle (COR = 0.44, *p* < 0.001). To further explore the relationship between TCA and prognosis of ESCC patients, we intersected the most relevant genes affecting TCA cycle activity in single cells and the differential genes identified by WGCNA, and identified 57 genes for further analysis (Figure 3A).

### Model construction and functional validation of ESCC risk models based on TCA cycling genes

The risk score model was constructed using the TCGA-ESCC training cohort. After initial screening of genes by univariate COX regression, LASSO regression further identified ATF3, MIDN, MAP3K8, PPP1R15A, CTTN, ARPC1A, CXCL14 and KRT19 as prognostically significant (Figure 3B), which were used to construct the model. The risk score for each sample was calculated as follows: $riskscore=\sum_{n=i}^{k}\left(Coef_{i}Expi\right)$ Based on the median value, patients were divided into the high- and low-risk groups. Of the eight model genes, five were risk factors and three were protective factors, and their specific weights are shown in Figure 3C. Furthermore, PCA and t-SNE assessments of these genes in the training and validation sets indicated that the ESCC patients could be grouped into training and validation cohorts (Figures 3D, E). We performed ROC curve analysis in both the training and test cohorts to further evaluate the predictive accuracy of the TCA cycle-based risk model. As shown in Figure 3F, the AUC values for predicting 1-, 2-, and 3-year survival in the training cohort were 0.814, 0.909 and 0.921 respectively, indicating that the TCA cycle genes can achieve prognostic stratification of ESCC patients. The AUC of the risk model for the validation cohort and the complete dataset were both greater than 0.7 (Figures 3G, H), thereby confirming the above findings. In the independent GSE53624 dataset, the AUC of the prognostic model for 1-, 2- and 3-year survival were 0.55, 0.588 and 0.591 respectively (Figure 3I). Although the values were less than optimum, they still confirm the predictive ability of the risk model. Furthermore, the high-risk group showed worse prognosis in the training, validation and entire TCGA cohort (Figures 3J–L; *p* < 0.05), and this result was validated in the GSE53624 cohort (Figure 3M). A nomogram was constructed using the TCA cycle risk score and clinical parameters (Supplementary Figure S1A). DCA and consistency index showed that the clinical value of the nomogram was significantly better than the individual clinical indicators, indicating a net benefit of using the nomogram for predicting patient prognosis in a clinical setting (Supplementary Figures S1B, C). Furthermore, the AUC of the nomogram for predicting 1-, 2-, and 3-year survival were 0.72, 0.754 and 0.902 respectively (Supplementary Figures S1D–F), which were significantly higher compared to the individual clinical predictors that comprised the model. Taken together, the TCA cycle risk score can accurately predict the prognosis of ESCC patients.

### Clinicopathological analysis of the TCA cycle-related risk model

**FIGURE 4:**
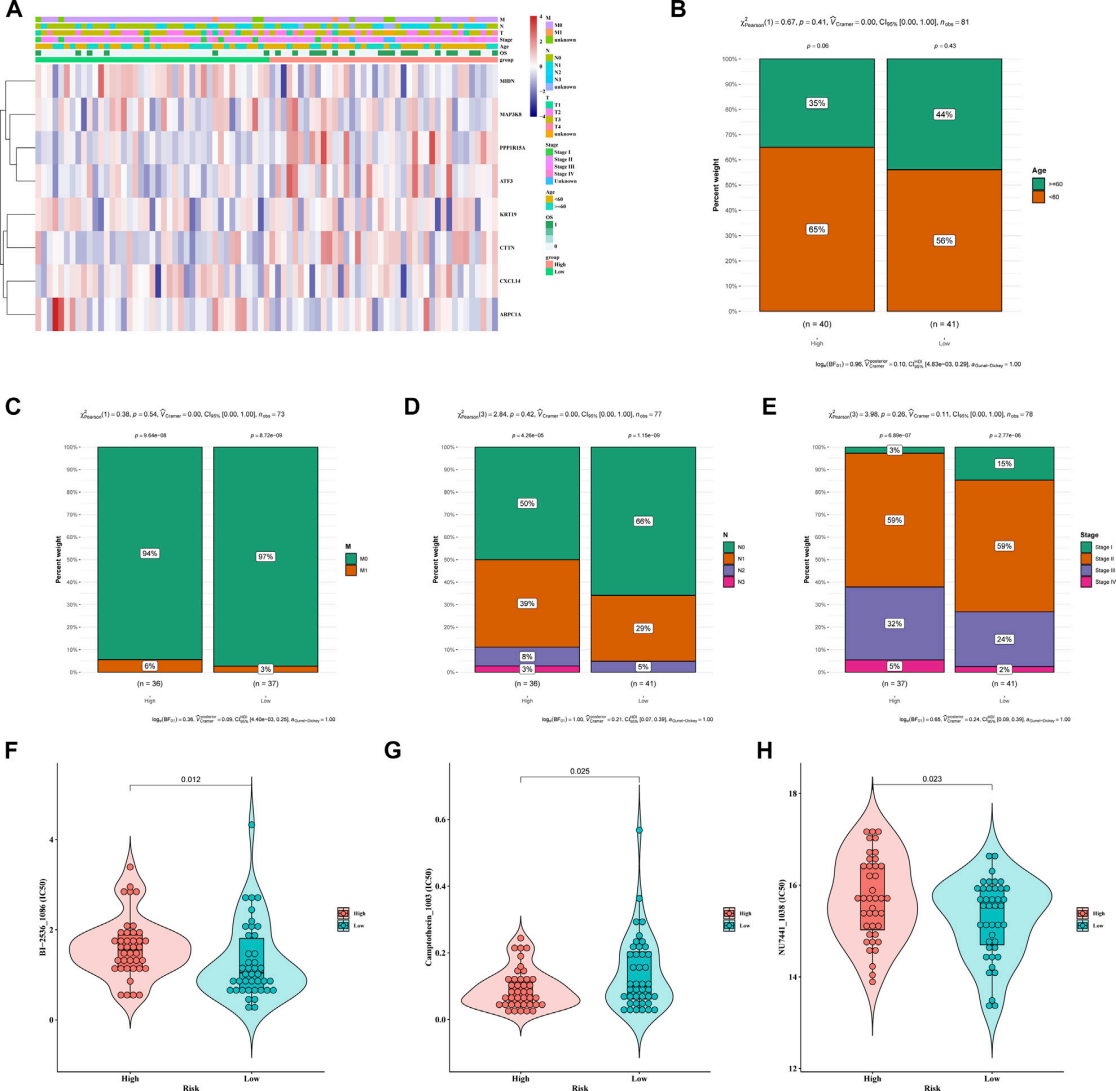
Clinicopathological analysis of different risk groups. **(A)** Heatmap of different clinical features of the two risk groups. **(B–E)** Distribution of the risk groups in **(B)** age, **(C)** M stage, **(D)** N stage and **(E)** TNM stage **(F–H)**. IC50 of the high-risk and low-risk groups to **(F)** BI-2536, **(G)** camptothecin and **(H)** NU7441.

To further elucidate the clinical relevance of the TCA cycle risk model in ESCC, we compared the clinical parameters between the high-risk and low-risk groups. As shown in Figure 4A, there were significant differences (*p* < 0.05) in the age, M classification, N classification, and TNM stage between the two groups. Patients in the high-risk group were younger, and had lower M-stage, higher N-stage and higher TNM stage (Figures 4B–E). We also compared the chemosensitivity of the risk groups, and identified BI-2536, camptothecin and NU7441 as possible drug candidates in the high-risk group (Figures 4F–H). This finding will aid in the selection of the most suitable drugs for clinical practice.

Analysis of mutation profiles revealed that missense mutations were most the common type. In addition, TP53, TTN and KMT2D were the top 3 most frequently mutated genes in ESCC, and C>T was the most common base pair substitution (Supplementary Figure S1A). The representative gene variants in the high-risk and low-risk groups are shown in Supplementary Figure S2B. We also analyzed the mutation status of the eight prognostic model genes, and found that the mutation frequencies of PP1R15A and CTTN were around 1% (Supplementary Figure S2C). Furthermore, analysis of the top 25 genes showed significant mutational correlations between TP53 and SMARCA4, PIK3CA, NOTCH1 and KMT2D, as well as between TTN and FMN2 (Supplementary Figure S2D). The combined prognostic impact of the risk score and the tumor mutational burden (TMB) was also assessed. As shown in Supplementary Figure S2EA, patients with higher TMB levels had a significantly worse prognosis.

### Association of the TCA cycle risk model with immune infiltration and drug sensitivity

**FIGURE 5:**
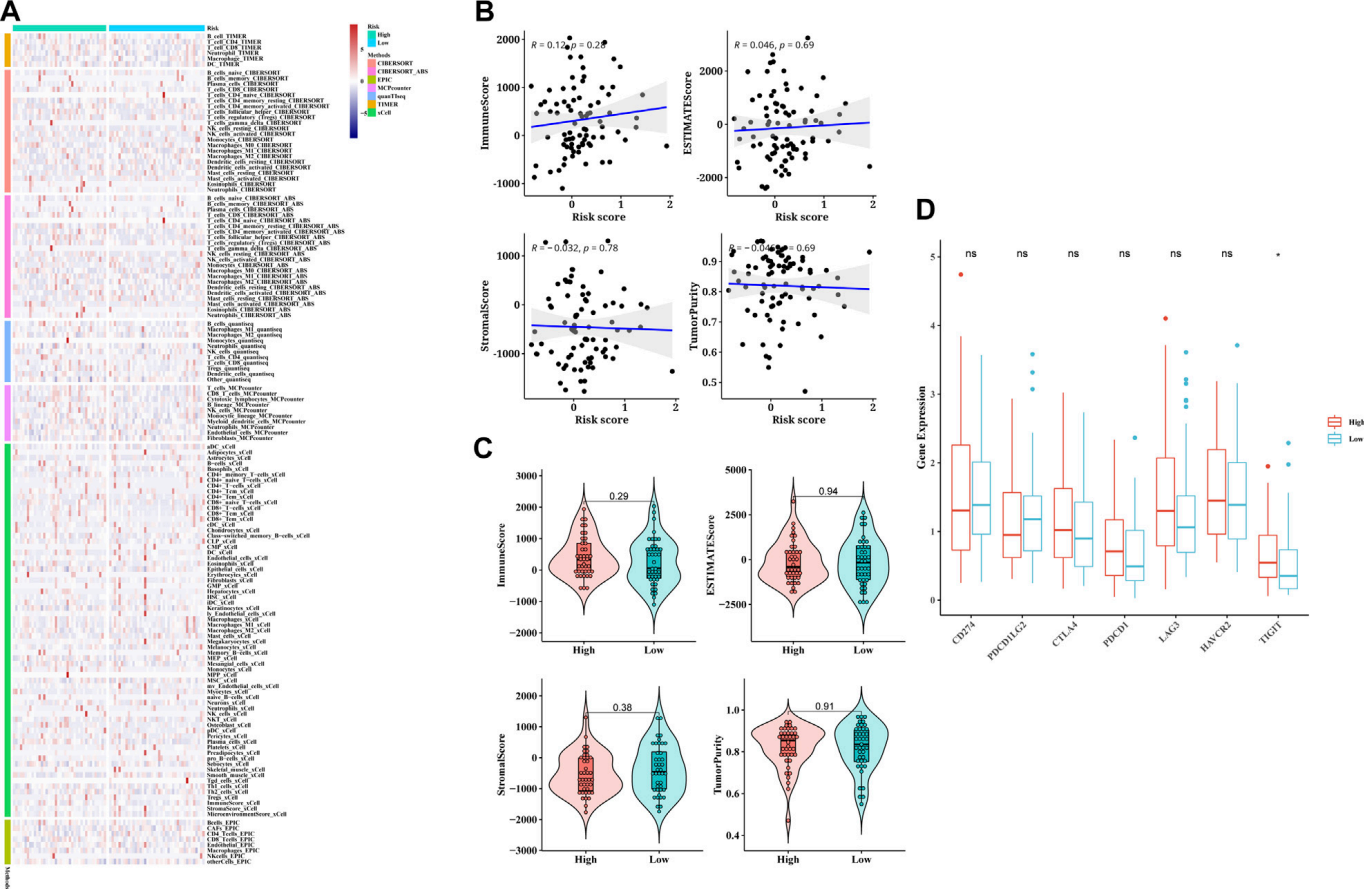
Correlation of TCA cycle-based risk score with immune cell infiltration and immune checkpoint expression in ESCC patients. **(A)** Correlation plots of 22 tumor-infiltrating immune cells (TICs) in different patients was mapped by the seven algorithms. **(B)** Scatter plots of linear correlation between tumor purity and TCA cycle risk score. **(C)** Violin plots showing the distribution of immune score, ESTIMATE score, Stromal scores, and tumor purity in the TCA cycle high-risk and low-risk groups. **(D)** Box plot showing the difference in the expression of immune checkpoint genes between the two risk groups.

**FIGURE 6:**
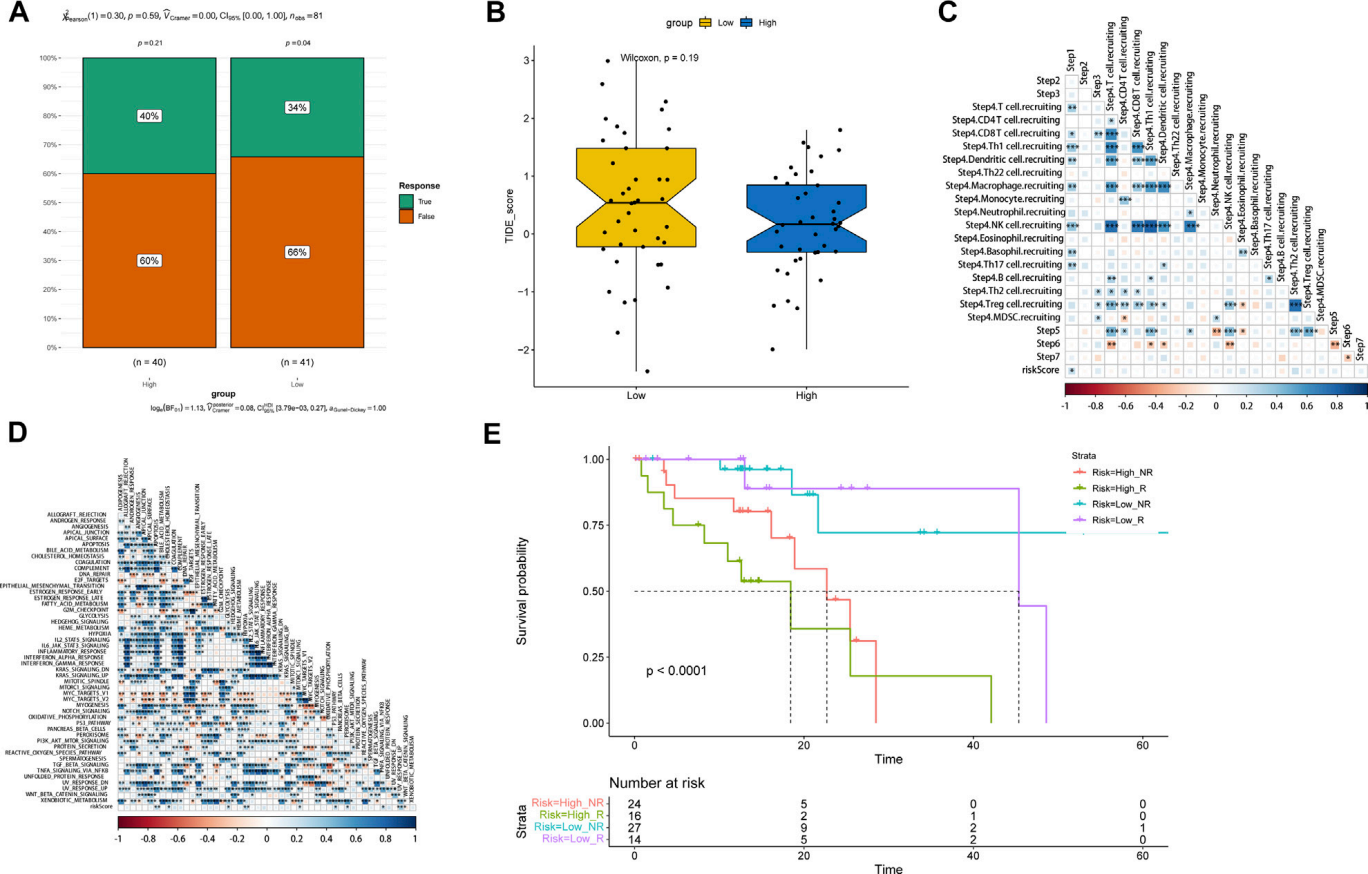
Evaluation of the predictive performance of TCA cycle risk score. **(A)** The ratio of response to immunotherapy of the risk groups. **(B)** Distribution of TIDE scores in the risk groups. **(C)** Heat map of the correlation between risk score and immune cell-related gene expression. **(D)** Heat map of the correlation between risk score and immune-related pathways. **(E)** Survival curves of patients stratified by the response to immunotherapy combining with TCA cycle risk score.

The CIBERSORT algorithm was used to determine the relationship between risk score with tumor immune infiltration. As shown in Figure 5A, immune infiltration was higher in the low-risk group compared to the high-risk group, except uncharacterized cells, common lymphoid progenitor cells and M2 macrophages. The correlation analysis of the risk score and multiple immune infiltration scores also showed greater tumor purity in the high-risk score group (Figure 5B). Furthermore, the low-risk group displayed higher stromal score, immunological score, and ESTIMATE score, suggesting greater immunogenicity (Figure 5C). The expression levels of different immune checkpoint-related proteins in the two groups were also compared. As shown in Figure 5D, TIGIT was significantly upregulated in the high-risk group (Figure 5D), which is indicative of an immunosuppressive TME. Taken together, the poor immune infiltration in ESCC may be associated with aberrant TCA cycle activity. To assess the performance of the TCA risk score in predicting immunotherapy response, we examined susceptibility to immunotherapy in different subgroups and found that patients in the high-risk group were more sensitive to immunotherapy (Figure 6A). As shown in Figure 6B, the high-risk group had lower TIDE scores, indicating better response to immunotherapy. In addition, we found that the risk score was positively related to step 1 of tumor immune process (Release of cancer cell antigens), and the risk score was also positively correlated to the apoptosis, cholesterol homeostasis, glycolysis, hypoxia, mTORC1 signaling, p53 pathway, peroxisome, TNFA signaling via NFKB, and UV response pathways (Figures 6C, D). Finally, survival analysis showed worse prognosis in the high-risk group with positive immunotherapy response (Figure 6E).

### CTTN is a potential oncogene for ESCC

**FIGURE 7:**
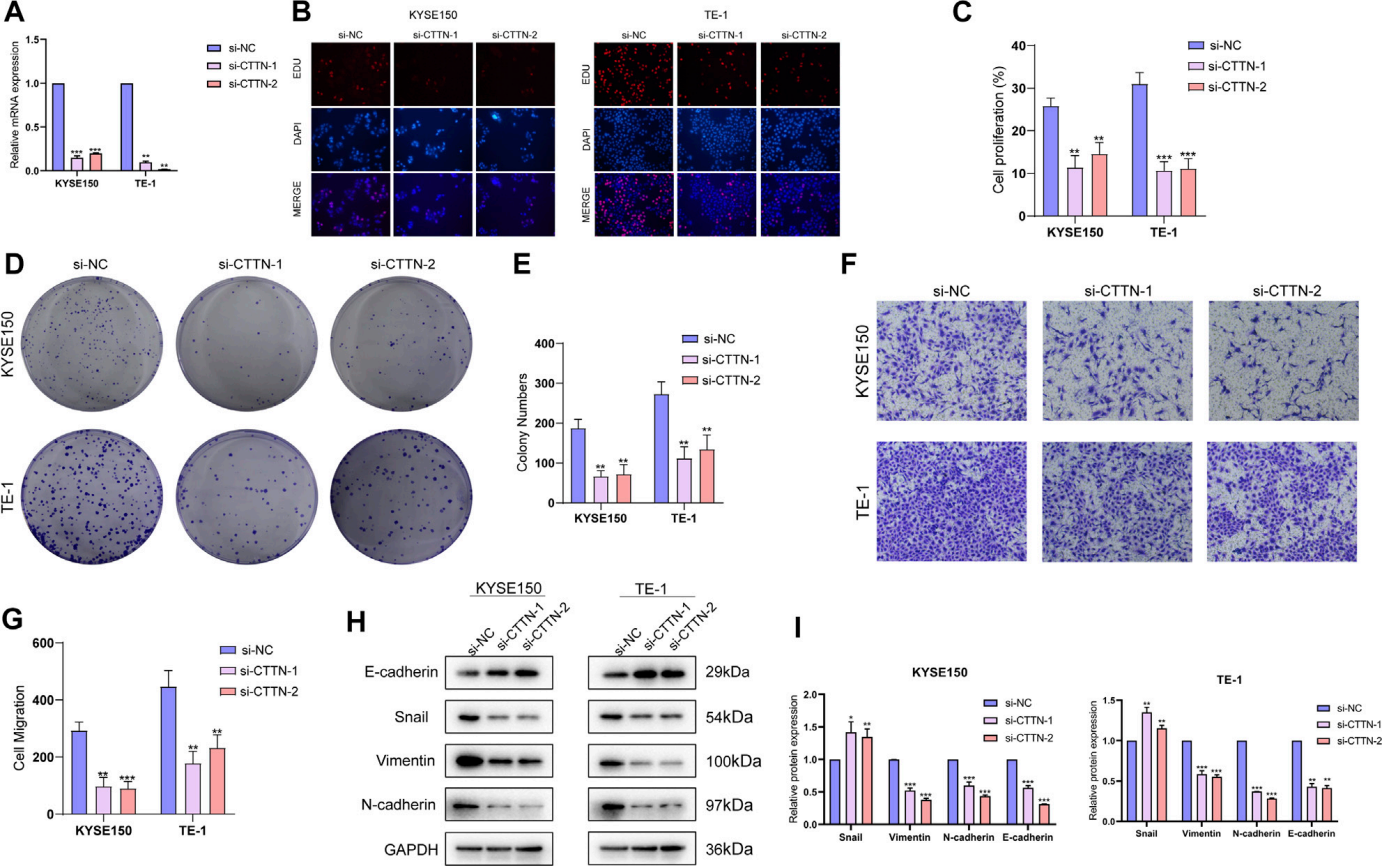
Functions of CTTN in the ESCC cells. **(A)** The relative expression levels of CTTN mRNA in KYSE150 and TE-1 cells transfected with si-NC, si-CTTN-1 and si-CTTN-2. **(B)** Representative fluorescence images showing EdU assays in the si-NC, si-CTTN-1 and si-CTTN-2 groups. **(C)** The proliferation rates in the groups. **(D)** Representative images of colonies formed by cells transfected with si-NC, si-CTTN-1 and si-CTTN-2. **(E)** Number of colonies in the groups. **(F)** Representative images of transwell assays showing invasion of cells transfected with si-NC, si-CTTN-1 and si-CTTN-2. **(G)** Proportion of invading cells in the groups. **(H)** Protein expression of E-cadherin, Snail, Vimentin and N-cadherin in the groups. **(I)** The quantification of protein levels.

To further elucidate the biological significance of the TCA-related genes in ESCC, we selected cortactin (CTTN), a key gene in risk score, for further analysis. The CTTN gene was successfully knocked down in the KYSE150 and TE-1 cell lines using specific siRNA sequences (Figure7A). The EdU assay showed that lower fluorescence intensity of cells transfected with si-CTTN-1/si-CTTN-2 compared to the si-NC group, indicating that CTTN influences proliferation of ESCC cells (Figures 7B, C). Consistent with this, the CCTN-knockdown cells formed markedly fewer colonies compared to the control cells (Figures 7D, E). Furthermore, knockdown of CTTN in the ESCC cells significantly decreased their invasiveness in the Transwell (Figures 7F, G). Since the high-risk score was positively correlated to EMT pathways, we also analyzed the expression levels of EMT-related proteins. CTTN knockdown upregulated the epithelial marker E-cadherin, and downregulated the mesenchymal markers Snail, Vimentin and N-cadherin in the ESCC cells (Figures 7H–I). These findings suggested that CTTN promotes invasion of ESCC cells by inducing their EMT.

## Discussion

ESCC is a major public health challenge worldwide. Although the prognosis of ESCC has improved in recent years with development of multimodality approaches, there is still lack of effective treatment approach for ESCC (Kakeji et al., 2021; Wang et al., 2022). Conventional clinical parameter such as TNM stage is often used to predict prognosis of ESCC (Marom, 2022). However, due to the significant tumor heterogeneity, the predictive accuracy of it is limited (Mahar et al., 2018). Therefore, there is an urgent need to identify new prognostic biomarkers and construct an effective model to predict patient prognosis and guide clinical treatment. The TCA cycle is a key metabolic pathway, that is, frequently dysregulated during tumor development (Anderson et al., 2018). Therefore, biomarkers based on the TCA cycle may have potential in prognostic prediction in ESCC.

Metabolic reprogramming is a hallmark of tumorigenesis (Martínez-Reyes and Chandel, 2021), and its underlying pathways and mechanisms are increasingly being explored as potential therapeutic targets for cancer (Hay, 2016). Recent studies have shown that the metabolism reprogramming of ESCC is conducive to tumor progression and chemoresistance (Chu et al., 2020; Liu et al., 2020). Given that the TCA cycle plays a crucial role in cellular energy metabolism, we analyzed the expression profile of TCA-related genes in ESCC at the population and single cell level, and identified key prognostic genes. A risk score model was constructed based on these genes to predict the prognosis of ESCC patients. The TCA-related risk score was correlated to the immune infiltration, immunotherapy response, chemoresistance and mutational burden in ESCC, and therefore has the potential to facilitate individualized clinical treatment plans. Bioinformatics analysis showed that the genes comprising the risk score model were significantly enriched in EMT-related pathways. Furthermore, knockdown of CTTN, a key gene of the risk score model, inhibited the malignant potential of ESCC cell lines *in vitro*. A previous study showed that downregulation of RNF128 induced EMT and stemness in melanoma cells through CD44 and CTTN ubiquitination (Wei et al., 2019). We have shown for the first time that CTTN promotes EMT and invasiveness of ESCC cells, which provides a new perspective to the relationship between metabolic abnormalities and EMT.

However, several limitations of this study also need to be considered. First, only a few ESCC datasets have complete clinical information (especially survival information) and gene expression data, which affect the reliability of the results. Therefore, the present findings will have to be validated further on independent datasets. Secondly, genetic differences among different ethnic groups may have an impact on the results, and should be taken into consideration in future studies. Thirdly, most of the genes in the risk model have not been previously associated with ESCC, and the correlation between these genes and esophageal cancer need to be investigated further. Finally, it remains to be elucidated whether this model can be applied to esophageal adenocarcinoma or even esophageal cancer in general.

In conclusion, we constructed a TCA cycle-based risk score to evaluate the prognosis, immune infiltration, immunotherapy response, and chemoresistance of ESCC patients. The risk score achieved good predictive performance in several independent datasets. CTTN, a key gene of the risk score model, affects the proliferation, invasion and EMT of ESCC cells, thus providing new insights into the relationship between metabolic abnormalities and EMT.

## Data Availability

The original contributions presented in the study are included in the article/Supplementary Material, further inquiries can be directed to the corresponding authors.
