# Supplementary figures and images for: A tricarboxylic acid cycle-based machine learning model to select effective drug targets for the treatment of esophageal squamous cell carcinoma
Source: Front Pharmacol. 2023 Jun 13;14:1195195. doi: 10.3389/fphar.2023.1195195 (PMC10294223; doi:10.3389/fphar.2023.1195195)

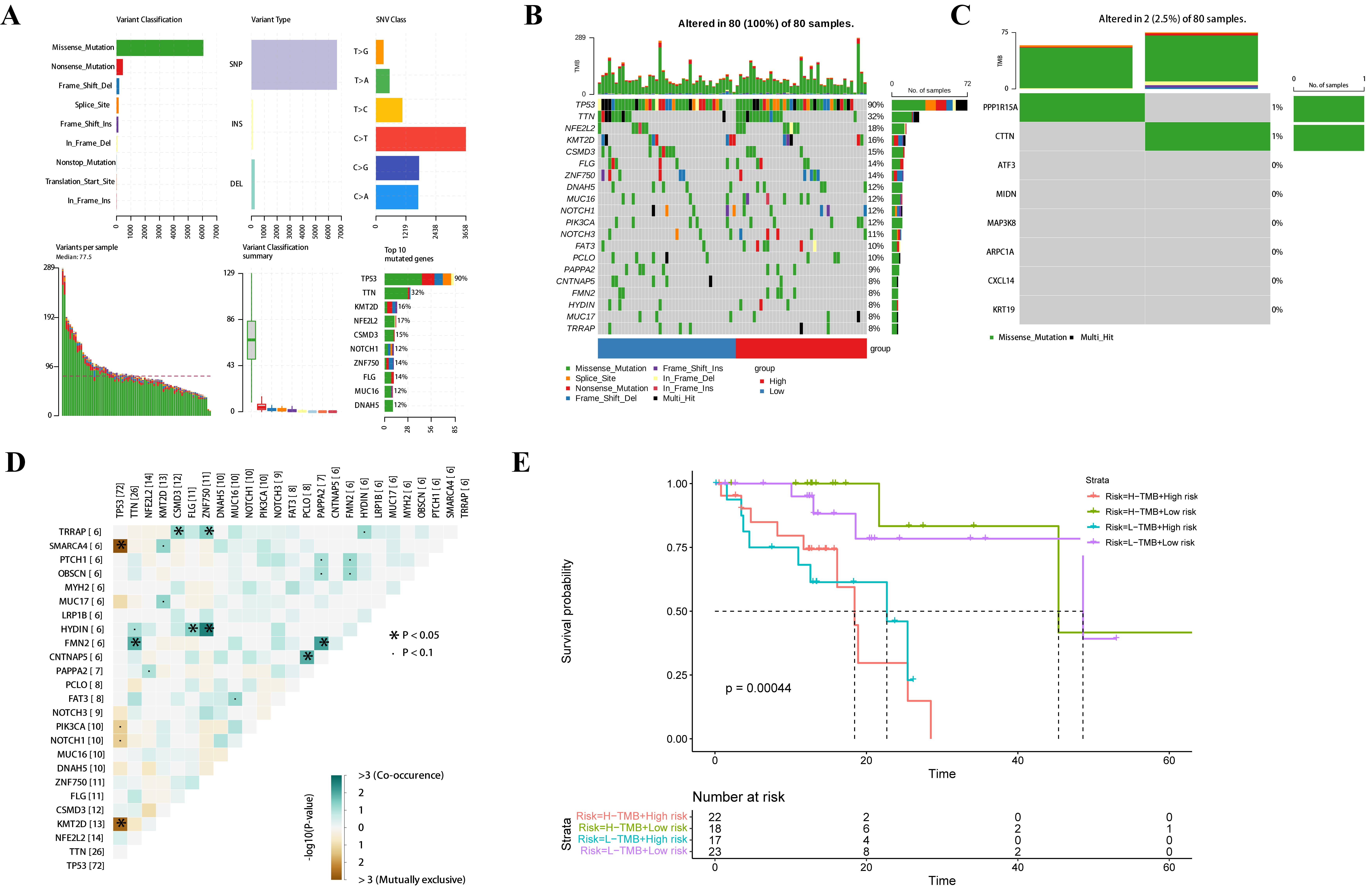

Supplement: Supplementary file 1 [file Image2.TIF]

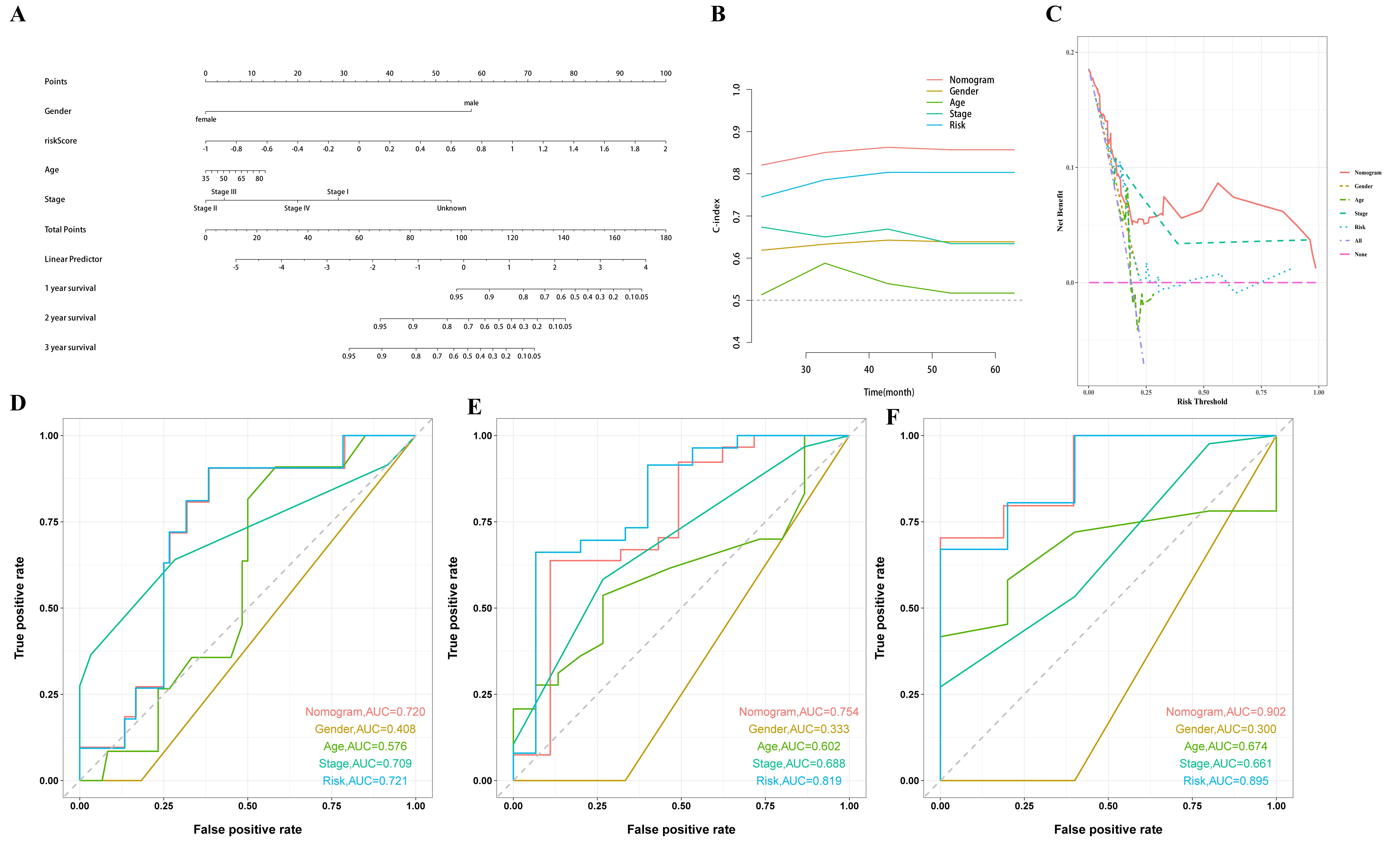

Supplement: Supplementary file 2 [file Image1.TIF]
